# Diode versus CO_2_ Laser Therapy in the Treatment of High Labial Frenulum Attachment: A Pilot Randomized, Double-Blinded Clinical Trial

**DOI:** 10.3390/ijerph17217708

**Published:** 2020-10-22

**Authors:** Gian Luca Sfasciotti, Francesca Zara, Iole Vozza, Veronica Carocci, Gaetano Ierardo, Antonella Polimeni

**Affiliations:** Department of Oral and Maxillo-Facial Sciences, Sapienza University of Rome, 00161 Rome, Italy; gianluca.sfasciotti@uniroma1.it (G.L.S.); iole.vozza@uniroma1.it (I.V.); veronica.carocci1@gmail.com (V.C.); gaetano.ierardo@uniroma1.it (G.I.); antonella.polimeni@uniroma1.it (A.P.)

**Keywords:** labial frenulum, frenulectomy, laser therapy, Diode laser, CO_2_ laser, pediatric dentistry

## Abstract

*Background:* The labial frenula are triangular plicas departing from the alveolar mucosa and attaching themselves at different heights of the gingiva. Sometimes a high attachment can determine a gingival recession. The most suitable surgical resolution is the use of laser devices. The aim of this study was to compare the labial frenulectomy through the use of Diode and CO_2_ laser techniques in pediatric patients with a high labial frenulum attachment, clarifying at the same time the preventive role of the surgical treatment to avoid further recession. *Methods:* A pilot randomized, double-blinded clinical trial was conducted to compare both the surgical advantages and the preventive treatment of laser technology using two different wavelengths within a population of pediatric patients with a high labial frenulum attachment. Different parameters intra and post-surgery were taken into account (Bleeding, Wound Healing, Gingival Recession, Periodontal pocket and Numerical Scale Value for pain) to compare Diode versus CO_2_ laser therapy. *Results:* Although both the laser devices provide a good performance in the post-operative period, the Diode laser shows better results (*p* < 0.001) in three of the five parameters evaluated. *Conclusions:* From the results it was found that the Diode Laser device is more suitable compared to the CO_2_ device.

## 1. Introduction

The labial frenula are anatomical structures found in the upper and lower vestibule of the oral cavity: they are triangular mucous plicas extending, in a sagittal direction, from the soft tissues and layering of the alveolar processes to the submucosa of the cheeks and lips. Histologically, they are constituted from connective tissue with a high percentage of collagen and a low percentage of adipose, vascular and nervous tissue; anatomically, both the orbicular and facial muscles can contribute to the structure of the labial frenula [1,2]. The muscles enable the frenula to regulate the facial structure development providing, in this way, the right support and stability, especially for the lips. Hence, possible alterations of the frenula can cause diastema, bone loss for muscle traction, limitations in lip mobility and gingival recession [3]. Regarding this latter alteration, it is important to understand how a high attachment of the lower labial frenula can cause the recession of the gingival margin accompanied by a detachment of the free gingiva from the dental surface during the distraction of the lower lip [4]. This detachment problem together with the ischemia of the tissues is a positive sign for the well-known pull-syndrome [2,4,5,6].

The pull-syndrome is common both in the upper and lower jaw, but the vector of forces is more harmful in this latter case [4]. In literature there are not any studies that show specific correlations between pull-syndrome and gingival recession or comparable epidemiological data concerning the onset of gingival recession in pediatric patients [7]. It seems that 20% of children are affected by gingival recession, which usually is not diagnosed before the gingiva completes its maturation at around 10 years of age [8]. The only clear evidence is that the gingival recession depends on several determinant or predisposing factors, such as high labial frenula attachment, and the incidence of this problem varies based on age, country, race and oral health conditions [9,10,11,12].

The oral surgery treatment is suggested both when the recession is already present or when atypical anatomical characteristics can determine further complications of it. The surgical approach of the lower labial frenulum is mandatory when it has an effect during teeth brushing maneuvers or in case there is a positive sign of pull-syndrome [13]. The plaque and bleeding index are above average when there is a high labial frenulum attachment which influences, negatively, the retention of plaque and the insurgence of gingivitis or gingival recession [14]. The oral surgery approach can be traditional with a scalpel, electrosurgery or with a laser, the use of which is broadening out and succeeding in different branches of dentistry [15,16,17]. The advantages of the single kind of lasers are well-showed in literature but, now as then, there are few studies concerning a comparison amongst their surgical performance, especially for the mandibular labial frenectomies [18,19,20]. The outcomes obtained using laser surgery technology are excellent but new comparative studies are also necessary to help the clinicians in the choice of the most appropriate device.

In this view, the aim of our study was to compare the labial frenulectomy through the use of diode and CO_2_ laser techniques in pediatric patients with a high labial frenulum attachment, clarifying, at the same time, the preventive role of the surgical treatment to avoid further recession.

## 2. Materials and Methods

### 2.1. Study Design and Blinded Methodology

A pilot randomized, double-blinded clinical trial was conducted to compare both the surgical advantages and the preventive treatment of laser technology with two different wavelengths in pediatric patients with a high labial frenulum attachment. This study used a randomized controlled experimental design based on the guidelines recommended by the Consolidated Standard of Reporting Trials—CONSORT 2010. The study protocol complied with the Guidelines for Good Clinical Practice, according to the Declaration of Helsinki (1975). Regarding the ethical approval, the study received the protocol number (0685) from the Institutional Review Board of the Department.

All patients enrolled were randomly divided in two groups for the treatment with Diode and CO_2_ laser device, respectively. In conformity with this clinical, randomized, double-blind study, neither the oral surgeon nor the patient were aware of the group to which they had been assigned. In addition, a different dental practitioner performed the follow-up for the next evaluations.

### 2.2. Randomization

The randomization sequence was generated using an online sequence generator [21]. A block randomization was implemented and 2 blocks of 13 non-unique per set were generated. All the participants were selected randomly regarding the equal distribution of gender

### 2.3. Dimension of Sample and Inclusion Criteria

Twenty-six patients were recruited by the Oral and Maxillo-Facial Sciences Department, Pediatric Dentistry Unit, University Hospital Policlinico Umberto I, “Sapienza” University of Rome from March 2018 to November 2019.

The inclusive criteria were: age between 7 and 12 years old, positive pull-syndrome test, no overlaps in mandibular incisors, no erosive tooth wear (score 0, using Basic Erosive Wear Lingual Tooth (BEWE), presence of recession (class I according to Miller scale), Clinical attachment loss (CAL stage I and II according to what were explained in the World Workshop 2017), no bad habits, no previous labial frenulectomy, no systemic diseases and signed consent by the legal guardian of each subject [22,23,24]. The exclusion criteria were: negative pull-syndrome test, presence of overlaps in mandibular incisors, presence of recession (class II-III-IV), CAL (stage III-IV), tooth wear (score 1-2^a^-3^a^), bad habits, previous labial frenulectomy, systemic diseases and no signed consent by the legal guardian of each subject [22,23,24]. 

In accordance with the aim of the study, to compare the performance of the two laser devices and the gingival height after operation, the following parameters were considered: Bleeding, Wound healing, Gingival Recession, CAL and Pain [23,24,25,26]. 

Different scores (1–2–3) were assigned to each parameter to simplify the statistical analysis:

- Bleeding was evaluated through the Ainamo and Bay blood index BI [24]. However, it was estimated: score 1 (absence of bleeding); score 2 (minimal bleeding); score 3 (copious bleeding) [25].
- Wound healing was clinically evaluated considering the residual amount of fibrin after oral surgery treatment. Unfortunately, there are no evaluative scales about wound healing process, hence according to what is explained in the book (Oral Wound Healing), it has been estimated: score 1 (wound totally covered by fibrin); score 2 (wound partially covered by fibrin); score 3 (complete healing without fibrin) [26].
- Gingival Recession was clinically evaluated through the use of a periodontal probe (P.C.P 15). Only patients with recession class I were considered, and it has been estimated: score 1 (0 to 1 mm of recession) score 2 (2 mm of recession) score 3 (3 mm of recession) [23].
- Clinical attachment loss was clinically evaluated through the use of a periodontal probe (P.C.P 15). Furthermore, for clinical attachment, only patients with stage I and II were considered and it has been estimated: score 1 (0–1 mm of probing); score 2 (2–3 mm of probing); score 3 (4 mm of probing) [24].
- Pain was calculated thought the use of the Numerical Rating Scale (NRS) which evaluation interval is from number 0 to 10.

Plaque index (Silness–Löe PI and O’leary PI) and Periodontal Phenotype by Muller et al. were also evaluated to understand if the presence of plaque and of a thin gingival phenotype could have negatively influenced the statistical results [27,28]. Even if the Silness–Löe PI and O’leary scale was considered during the recruitment process, it estimated three scores to simplify and standardize the values with the other parameters mentioned above: score 1 (absence of plaque); score 2 (poor presence of plaque); score 3 (copious presence of plaque). According to the Silness–Löe PI and O’leary scale, the evaluation of these three different scores was done considering the presence of plaque in the whole mouth, and also the molar teeth were included. On the other hand, according to what Muller explains, the different phenotype evaluated were: Cluster A1 (Thin) with Thin Gingival Thickness (GT), narrow width of Keratinized Tissue (KT) and slender teeth; Cluster A2 (Thin) with thin GT, wide KT and slender teeth; Cluster B (Thick) with thick GT, wide KT and quadratic teeth [28].

Most of recruited patients had no copious presence of plaque and a gingival phenotype cluster A2 and B (Table 1). Hence, these two conditions did not negatively influenced the results obtained.

### 2.4. Surgical Protocol

All the oral surgery treatments are carried out setting up the two devices following the protocol of the study (Table 2). The same oral surgeon did the interventions, after the administration of both topic anesthesia (Lidocaine 15%) for 4 min and following injection of 0.9 mL of local anesthesia without a vasoconstrictor.

**Figure 1:**
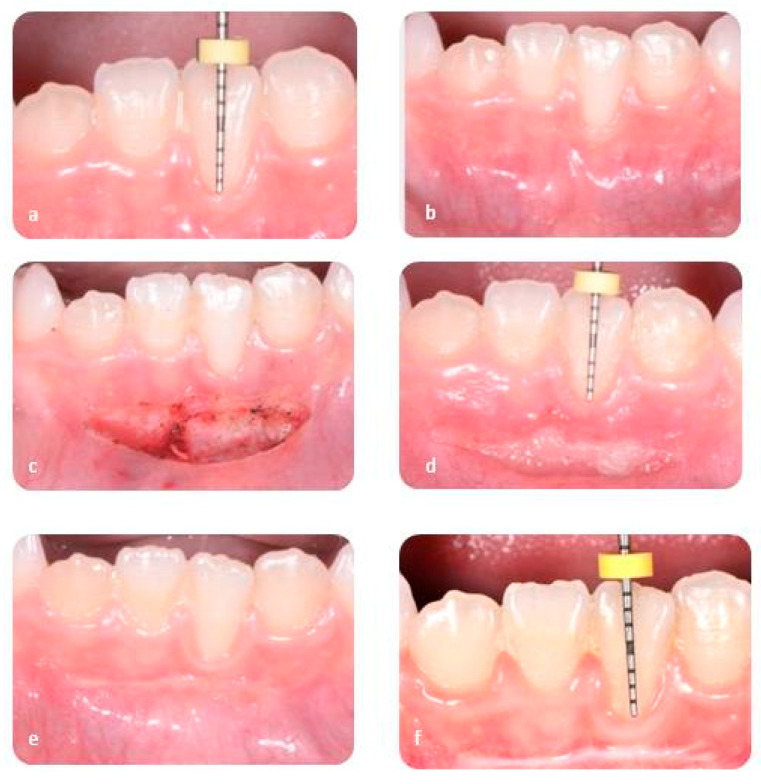
Diode laser: pre-operative evaluation (**a**,**b**), surgical wound (**c**), follow-up after seven days (**d**), follow-up afeter thirty days (**e**), follow-up after six months (**f**).

**Figure 2:**
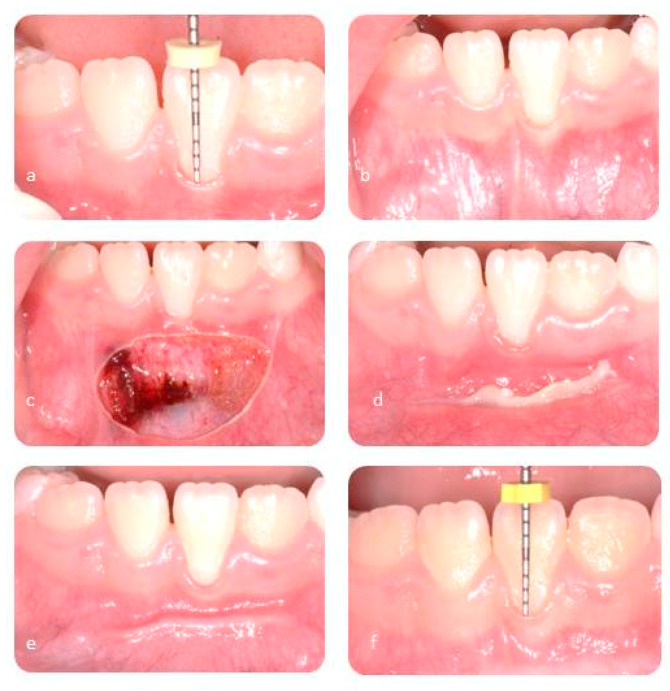
CO_2_ laser: pre-operative evaluation (**a**,**b**), surgical wound (**c**), follow-up after seven days (**d**), follow-up afeter thirty days (**e**), follow-up after six months (**f**).

The traction of the lip muscles was achieved through the use of a gauze and it was held for the entire duration of the intervention. The extremities of the two laser devices were kept upright on the labial frenulum and were moved slowly with oscillatory movements. With this technique it is possible to avoid possible necrotic phenomena. In all cases the incision only involved the superficial part of mucosa layers without removing periosteal adhesion. No sutures were required. The intervention with the Diode laser has taken about 550 s; on the other hand, the duration of treatment with CO_2_ has been about 300 s (Figure 1 and Figure 2).

### 2.5. Clinical Case

After the operation, a spray mouthwash was administered (chlorhexidine 0.2%), for 7 days after the operation. For each patient, a diary was distributed with the NRS and queries concerning the possible consumption of painkillers which were not directly administered. 

No patients required a pharmacological protocol for the control of anxiety before surgery.

### 2.6. Statistical Methodology

Data were collected in a database using Excel (Microsoft, Redmond, WA, USA). The following analysis and the calculation of *p*-value was done through a standard statistical analysis software (version 20.0, Statistical Package for the Social Sciences, IBM Corporation, Armonk, NY, USA).

Descriptive methods were used to summarize patients’ demographic information and the parameters: Bleeding, Wound Healing, Gingival Recession, CAL and pain. In addition, differences between pre-treatment and reentry data were analyzed with a paired t-Student test.

## 3. Results

The average age of the sample was 9 years (M = 8.66; DS = 2.31) and all patients who had taken part in the study were included in a follow-up program. Considering the parameters included in this study, the follow-up times were designed in accordance with different clinical and biological behavior. Indeed, Bleeding, Wound Healing, Gingival recession and CAL were evaluated at T1 (the day after), T2 (15 days after), T3 (30 days after) and T4 (6 months after). However, in accordance with a faster change of pain over the time, it was evaluated at T1 (24 h), T2 (48 h), T3 (72 h) and T4 (14 days after the operation) to better understand how the two different laser devices can influence it. 

Data related to descriptive methodology are explained in the following Table 3. Instead, Table 4 represents the paired t-test results of the parameters for both the Diode Laser and CO_2_ in the manner of mean differences which vary from T1 and T4 on average. It is important to remark that for wound healing, time periods have been chosen as T2 and T3 because the descriptive analysis showed more relevant data in these interval of time in order to see the mean differences after both the Diode Laser and CO_2_ treatment. 

For this kind of analysis, T1 was used as initial time period because T0 captured patients before treatment, so T0 was the initial state. In the table, SEM represents for sample estimation of mean differences and estimate the difference as the mean value of before/actual time of the treatment (T1) minus the mean value after six months of the treatment (T4). Hence, if SEM is positive, then one may interpret that after the treatment the outcome decreased since the first mean value is greater than the second one. Contrarily, a negative SEM value refers to an increase in the outcome regarding to the same logic.

In accordance with what the Table 3 shows, it is found that after six months, mean differences for both the Diode Laser and CO_2_ are statistically significant:

- In the manner of patients’ bleeding. In particular, after six months passed after the treatment, using CO_2_ caused fewer bleeding cases on average compared to the Diode Laser treatment. Specifically, we observed this impact on female patients more significantly rather than male patients
- In the manner of wound healing there is very strong evidence that after the treatment with the Diode Laser, patients’ wounds showed fast healing with respect to CO_2_ treatment
- In the manner of gingival recession, there is very strong evidence that the treatment with Diode Laser causes reducing patients’ gingival recession that is, on average, significantly better than the CO_2_ treatment. In particular, gingival recession of male patients decreases fast compared to female patients with Diode Laser treatment, whereas CO_2_ treatment has a better outcome on the reduction of gingival recession in female patients rather than male patients.
- In the manner of *CAL*, it can be said that after six months of the treatment with Diode Laser, the improvement of *CAL* is statistically significant and firmly reducing the periodontal pocket occurrence, whereas, there is no significant finding related to *CAL* after CO_2_ treatment
- Lastly, in the manner of *NRS*, it can be said that both methods reduce the pain very efficiently, Diode Laser treatment removes the pain faster than CO_2_ treatment. In fact, during the first fourteen days after the treatment, there was a reduction by 89% of pain in the group where it was used diode laser technique. On the other hand, in the same time rate, the reduction of pain was by 65% in the group where a CO_2_ laser was used. Notably, it is found that male patients got over the pain with Diode Laser easier than female patients, whilst CO_2_ treatment helped to remove the pain more effectively on female patients with respect to male patients.

## 4. Discussion

Most of articles in literature are, unfortunately, a long way from an in-depth comparison between two laser devices in the treatment of a high labial frenula attachment. Hence, nowadays it is still a challenge to decide the choice between different kinds of laser devices and the consequent advantages and disadvantages for each one of them. In view of this, the aim of our study was a comparison of two different laser devices in the treatment of a high labial frenula attachment. Unfortunately, a direct correlation between our results and other studies was not possible because, even though many of them have considered the same as our parameters, they have only designed a comparison between scalpel and laser and not between two different laser devices. For this reason, the unique sure affirmation, which can be inferred from literature, is that the laser technique shows better results compared to the traditional technique with a scalpel. Indeed, laser devices provide several advantages such as less post-operative discomfort and a better wound healing process in the post-operative period [29,30,31,32,33,34]. 

A recent meta-analysis by Protásio et al. (2019) shows, after a detailed lecture of 1639 articles, how only six articles were considered suitable in comparing the scalpel and laser technique in the treatment of labial frenula [29]. This meta-analysis reveals how bleeding cannot be estimated because each author evaluated it through the use of a different scales. On the other hand, less post-operative discomfort and no need for suture were estimated when a laser device was used [29]. Unfortunately, in literature there are no meta-analyses which provide for a comparison between two different kinds of lasers. Indeed, also in the study followed by Calisir and Ege, was done an evaluation of the post-operative discomfort between scalpel and laser (Nd:Yag) in the treatment of frenula. Results show less post-operative discomfort when a Nd:Yag device was used compared to a scalpel [30].

Another interesting study, which was carried out by Seizgin et al., reveals how both conventional and laser-assisted frenectomy surgeries prevent the frenula reattachment when there is a periosteal horizontal incision [31]. The clinical parameters evaluated were the wound healing, pain, the distance between frenula attachment point and mucogingival junction, plaque index, gingival index and probing depth. Forty-eight patients were divided into three groups: the first one with conventional frenectomy operation (C group), the second one with diode laser-assisted frenectomy (L group) and the third one with diode laser-assisted frenectomy with added conventional horizontal incision on the periosteum (L + P group) [30]. The results suggest how the diode laser technique provides for a better post-operative patient’s perception of pain without significant differences in the distance between the frenula attachment point and mucogingival junction (FMGJ) [31]. Even though this study was designed in a similar way to our research, data reports a comparison between scalpel and diode laser, so it is unhelpful to evaluate the distinct performance of two different laser devices. Another study carried out by A. Uraz et al., shows valid results but unfortunately is a comparison between scalpel and diode laser treatment [32]. The clinical parameter considered were plaque index, gingival index, probing and CAL. Furthermore, in this case, the main results were that both conventional and diode laser techniques are efficient in gaining a good healing and an improvement of keratinized gingiva width (KGW), attached gingiva width (AGW) and attached gingiva thickness (AGT) recorded at follow-ups. However, the diode laser provides for less postsurgical patient discomfort with a reduction of pain and possible complications. The same conclusion was reached by Calisir and Ege; they designed a split-mouth randomized comparative study to evaluate the clinical outcomes of frenectomy performed with conventional or Nd:YAG laser surgery [31]. The latter was more suitable compared to the traditional technique in terms of enhanced patient comfort and lower pain levels [31]. The superiority of the diode laser on the traditional technique with a scalpel was demonstrated in another study where thirty patients were divided into three groups and all of them followed a surgical treatment for an aberrant frenum attachment [33]. The parameters intended were wound healing, operative time and post-operative discomfort. As is already anticipated in literature, the diode laser provided for a better wound healing and a reduction of both operative time and pain perception [33].

Another relatively recent meta-analysis dates back to 2018 and shows how laser therapy can help in terms of Width of Keratinized Tissue, Probing Depth and CAL when it is associated with the surgical treatment of the recession using a scalpel; at the same time root coverage and esthetics were not optimal [34]. Compared to the purpose of our study, it is clear how this meta-analysis is off-topic but, in any event, it is interesting to consider the different ways in which the application of laser can help the healing of wounds and an early creeping of the gingival margin. A good comparison concerning the treatment of upper frenula through three different lasers (CO_2,_ Er and Cr:YSGG) shows how the use of a CO_2_ laser provides for bloodless fields and shorter surgical times compared to the Er and Cr: YSGG [19]. This latter study, carried out by Pié-Sánchez J, also highlights that both Er and Cr:YSGG provide for faster wound healing [19]. Three studies show an early creeping attachment after frenuloplasty in a patient with a pathological labial frenulum; however, all of them report only a single case and moreover apply the traditional technique with a scalpel [14,35,36]. Studies like these ones provide for a good starting point concerning the necessity of a preventive surgery to avoid further complications of the recession, as discussed above, but at the same time they are not enough to understand the advantages and disadvantages amongst different kinds of treatment [32]. It is clear how preventive treatment and prophylaxis of the recession help in avoiding a possible aggravation, but the data show it is not sufficient to determine which kind of laser treatment offers the best results.

Considering the gaps present in literature, our study provides for a great comparison between the Diode and CO_2_ laser technique and, in particular, the effects of them in the treatment of high labial frenula attachment.

## 5. Conclusions

Despite the fact that this study provides for a great improvement in the knowledge about lasers with different wavelengths in the treatment of high labial frenula attachment, some limitations need to be underlined. Indeed, a larger sample size and information in relation to the thickness of frenula before and after oral surgery treatment should be introduced in further studies. In accordance with these points, further randomized studies are essential to improve more and more laser devices performances and to understand the efficacy of them in oral surgery treatments.

However, in relation to this article, it was found that the Diode Laser device is more suitable compared to the CO_2_ device in the treatment of high labial frenula attachment. The CO_2_ device is less suitable in terms of wound healing, CAL, gingival recession’s improvement and post-operative comfort. The higher discomfort of the CO_2_ laser is likely related to a deeper penetration of X-rays in the soft tissue. In addition, it is also important to remark that the clinician also needs more time to learn the correct application of a CO_2_ laser device.

On the whole, the Diode laser seems to be more appropriated in terms of biological outcome and ease of use with a reduction in intra-operative mistakes depending on the clinician.

## Figures and Tables

**Table 1:** Incidence of plaque and features of periodontal phenotype.

	Periodontal Phenotype			Plaque Index		
	A1	A2	B	1	2	3
CO_2_	1	7	5	10	0	3
Diode Laser	1	4	8	8	2	3

**Table 2:** Setting of the devices.

Type	Diode	CO_2_
Name	Raffaello Bio	Smart US20D
Wavelength	980-nm	10.600 nm
Power	2.5 W	4.5 W
Modality of pulse	Continuous	Super pulse wave
Frequency	1.000 Hz	80 Hz
Medical class/Laser	IIB/IV	IIB/IV
Number of sessions	Single surgery session	Single surgery session
Production site.	DMT Dental Medical Technology	Deka

**Table 3:** Descriptive statistic of parameters.

		Bleeding			Wound Healing			Gingival Recession			CAL			NRS		
		Mean	SD	SEM	Mean	SD	SEM	Mean	SD	SEM	Mean	SD	SEM	Mean	SD	SEM
Diode Laser	T0	1.15	0.55	0.15	3.00	0.00	0.00	1.69	0.63	0.17	1.46	0.66	0.18	0.00	0.00	0.00
	T1	1.62	0.65	0.18	1.69	0.48	0.13	1.62	0.51	0.14	1.46	0.66	0.18	3.62	2.96	0.82
	T2	1.31	0.63	0.17	1.92	0.28	0.08	1.62	0.51	0.14	1.46	0.66	0.18	1.54	1.56	0.43
	T3	1.15	0.55	0.15	2.54	0.66	0.18	1.00	0.00	0.00	1.15	0.38	0.10	0.08	0.28	0.08
	T4	1.15	0.55	0.15	2.77	0.60	0.17	1.00	0.00	0.00	1.08	0.28	0.08	0.00	0.00	0.00
	N	13	13	13	13	13	13	13	13	13	13	13	13	13	13	13
CO_2_	T0	1.00	0.00	0.00	3.00	0.00	0.00	1.38	0.77	0.21	1.31	0.63	0.17	0.00	0.00	0.00
	T1	2.00	0.58	0.16	1.92	0.49	0.14	1.38	0.77	0.21	1.31	0.63	0.17	3.38	2.26	0.63
	T2	1.38	0.51	0.14	1.92	0.28	0.08	1.23	0.60	0.17	1.23	0.44	0.12	1.38	1.33	0.37
	T3	1.00	0.00	0.00	2.23	0.93	0.26	1.08	0.49	0.14	1.23	0.44	0.12	0.23	0.83	0.23
	T4	1.00	0.00	0.00	2.54	0.97	0.27	1.00	0.41	0.11	1.15	0.38	0.10	0.00	0.00	0.00
	N	13	13	13	13	13	13	13	13	13	13	13	13	13	13	13

**Table 4:** Paired *t*-test results of the parameters (between T1 and T4).

		Diode Laser			CO_2_		
		*t*-Test	*p*-Value	SEM	*t*-Test	*p*-Value	SEM
Bleeding	Female	2.828	0.030 **	0.571	4.582	0.004 **	1
	Male	1.581	0.175	0.333	3.873	0.012 **	1
	Total	3.207	0.008 **	0.462	6.245	0.000 ***	1.000
Wound Healing	Female	−3.873	0.008 **	−0.714	−1.441	0.199	−0.429
	Male	−2.236	0.076 *	−0.500	−1	0.363	−0.333
	Total	−4.383	0.000 ***	0.616	−1.806	0.096 *	−0.385
Gingival Recession	Female	2.121	0.078 *	0.429	2.828	0.030 **	0.571
	Male	5	0.004 ***	0.833	1	0.363	0.167
	Total	4.382	0.000 ***	0.615	2.739	0.018 **	0.385
Periodontal Pocket	Female	2.827	0.031 **	0.572	1	0.356	0.143
	Male	1	0.363	0.167	1	0.360	0.165
	Total	2.612	0.017 **	0.384	1.477	0.165	0.154
NRS	Female	2.547	0.043 **	2.857	4.831	0.002 **	3.285
	Male	3.737	0.013 **	4.5	2.976	0.031 **	3.572
	Total	4.405	0.000 ***	3.615	5.409	0.000 ***	3.385

*, ** and *** indicate statistical significance for 1%, 5% and 10% the levels of confidence for rejection of the null hypothesis. SEM refers to sample estimate for mean of the differences.
